# Lapse in Institutional Animal Care and Use Committee Continuing Reviews

**DOI:** 10.1371/journal.pone.0162141

**Published:** 2016-09-08

**Authors:** Min-Fu Tsan, Michael Grabenbauer, Yen Nguyen

**Affiliations:** 1 McGuire Research Institute, Richmond, Virginia, United States of America; 2 Office of Research Oversight, Department of Veterans Affairs, Washington, District of Columbia, United States of America; Central South University, CHINA

## Abstract

The United States federal animal welfare regulations and the Public Health Service Policy on Humane Care and Use of Laboratory Animals require that institutional animal care and use committees (IACUCs) conduct continuing reviews of all animal research activities. However, little is known about the lapse rate of IACUC continuing reviews, and how frequently investigators continue research activities during the lapse. It is also not clear what factors may contribute to an institution’s lapse in IACUC continuing reviews. As part of the quality assurance program, the Department of Veterans Affairs (VA) has collected performance metric data for animal care and use programs since 2011. We analyzed IACUC continuing review performance data at 74–75 VA research facilities from 2011 through 2015. The IACUC continuing review lapse rates improved from 5.6% in 2011 to 2.7% in 2015. The rate of investigators continuing research activities during the lapse also decreased from 47.2% in 2012 to 7.4% in 2015. The type of IACUCs used and the size of animal research programs appeared to have no effect in facility’s rates of lapse in IACUC continuing reviews. While approximately 80% of facilities reported no lapse in IACUC continuing reviews, approximately 14% of facilities had lapse rates of >10% each year. Some facilities appeared to be repeat offenders. Four facilities had IACUC lapse rates of >10% in at least 3 out of 5 years, suggesting a system problem in these facilities requiring remedial actions to improve their IACUC continuing review processes.

## Introduction

The United States federal animal welfare regulations require that the institutional animal care and use committee (IACUC) conducts continuing reviews of animal research activities at appropriate intervals as determined by the IACUC, but not less than once per year [1]. The intent of this requirement is to provide information to the research facilities regarding all on-going animal research activities to ensure compliance. On the other hand, the United States Public Health Service (PHS) Policy on Humane Care and Use of Laboratory Animals requires that the IACUC continuing reviews of animal research activities be conducted not less than once every three years [2]. The Office of Laboratory Animal Welfare (OLAW) has interpreted this PHS Policy provision for triennial continuing reviews as a requirement for a “de novo” review, requiring a comprehensive review that satisfies all criteria for the approval of initial protocol reviews [3]. Thus, the purpose of continuing reviews appears to be threefold, namely, to inform the IACUC of the current status of the project, to ensure continued compliance with federal animal welfare regulations, PHS Policy, and institutional requirements, and to provide for re-evaluation of the animal research activities at appropriate intervals [3].

To comply with both the federal animal welfare regulations and the PHS Policy continuing review requirements, the Department of Veterans Affairs (VA) requires that IACUCs must review the conduct of all animal protocols annually. At the first and second anniversaries, the IACUC may review a standard form giving current basic information about the protocols. However, prior to the third anniversary, the IACUC must conduct a complete “de novo” review of the protocol [4].

Failure to conduct timely IACUC continuing reviews may result in suspension or termination of protocols, causing considerable interruption and disruption of animal research activities. However, despite the importance of timely IACUC continuing reviews, little is known about the lapse rate of IACUC continuing reviews and how frequently investigators continue research activities during the lapse. It is also not clear what factors may contribute to an institution’s rate of lapse in IACUC continuing reviews.

The VA Health Care System is the largest integrated health care system in the United States with 75 facilities conducting research involving laboratory animals in 2015. As part of the quality assurance program, VA has been collecting performance metric data, including IACUC continuing reviews, for animal care and use programs since 2011 [5].

In the current study, we analyzed VA IACUC continuing review data from 2011 to 2015. We report here the lapse rates in IACUC continuing reviews over a 5-year period and whether the size of animal research programs or the types of IACUCs used, has any effects on lapses in IACUC continuing reviews.

## Methods

### Data collection

As part of the VA quality assurance program, VA facilities conducting research involving laboratory animals were required to conduct regulatory audits of all active animal research protocols once every 3 years [6]. Audit tools were developed and research compliance officers were trained to use these tools to conduct audits throughout the year [7]. Approximately one third of all active animal research protocols were audited each year.

Results of the protocol regulatory audits conducted between June 1 and May 31 of each year were collected through a web-based system from all VA research facilities. Information collected included: IACUC and Research and Development Committee initial approval of animal research protocols; for-cause suspension or termination of animal research protocols; compliance with IACUC continuing review requirements; research personnel scopes of practice; and investigator animal research protection training requirements [5].

As this was a VA quality assurance project and it did not involve the use of laboratory animals or human subjects including collection of individually identified private information, no IACUC or Institutional Review Board review and approval was required [8].

### Data analysis

All data collected were entered into a computerized database for analysis. We used the analysis of ordered categorical data to determine the trend of changes from 2011 through 2015 [9]. This was performed using JavaStat ordinal contingency table analysis available at www.statpages.info. For the comparison of two means, the Student’s t test was used to determine the level of significance. A *p* value of < 0.05 was considered to be statistically significant. When multiple comparisons were required, post hoc analysis using Bonnferoni correction for multiple comparisons was performed [10].

## Results

### Lapse in IACUC continuing reviews

Table 1 summarizes the data on IACUC continuing reviews. The lapse rate of all protocols audited was 5.6% in 2011. It decreased in subsequent years and was 2.7% in 2015. Despite a spike of lapse rate in 2014 to 4.3%, there was a statistically significant trend of improvement from 2011 through 2015 (p = 0.0013 using analysis of ordered categorical data).

**Table 1:** Lapse in institutional animal care and use committee continuing reviews.

	2011	2012	2013	2014	2015	P-value
Number of facilities	74	74	74	74	75	
Total number of protocols	2,830	3,107	3,203	3,119	3,087	
Number of protocols audited	1,347	1,286	1,147	1,067	982	
Lapses in continuing reviews	75 (5.8%)^1^	53 (4.1%)	23 (2.0%)	46 (4.3%)	27 (2.7%)	0.0013
Continued research during lapse	-^2^	25 (47.2%)^3^	6 (26.1%)	12 (26.1%)	2 (7.4%)	0.0069

^1^ The numbers in parentheses were the percentages of the total number of protocols audited.

^2^ Not collected.

^3^ The numbers in parentheses were the percentages of the total number of protocols with lapse in institutional animal care and use committee continuing reviews.

In nearly a half of lapsed protocols, investigators continued animal research activities during the lapse in 2012 (2011 data not collected). As shown in Table 1, the rate of investigators who continued research activities during the lapse also significantly improved from 47.2% in 2012 to 7.4% in 2015 (*p* = 0.0069)

### Effect of the types of IACUC used

Based on the types of IACUC used, VA research facilities can be categorized into two groups, i.e., those using their own VA IACUCs and those using affiliate university IACUCs as their IACUCs of record. We analyzed our data to determine whether the type of IACUCs used had any effect on lapses in IACUC continuing reviews.

As shown in Table 2, on the average, approximately 60 facilities each year used their own VA IACUCs and 14 facilities each year used affiliated university IACUCs as their IACUCs of record. However, the type of IACUCs used had no effect on the facility’s lapse rate in IACUC continuing reviews (*p* = 0.5607, Student’s t test).

**Table 2:** Lapse in continuing reviews according to types of institutional animal care and use committee used.

IACUC Types	2011	2012	2013	2014	2015	Mean (±SD)
**VA IACUC**						
Number of facilities	61	61	60	60	60	60.4 (±0.5)
Lapse rates	5.7%	4.2%	2.1%	4.4%	2.3%	3.74% (±1.52%)^1^
**Affiliate IACUC**						
Number of facilities	13	13	14	14	15	13.8 (±0.8)
Lapse rates	3.8%	2.8%	1.5%	1.9%	5.6%	3.08% (±1.62%)

^1^p value: 0.5607 (VA IACUC vs. Affiliate IACUC).

### Effect of the size of animal research programs

We also analyzed our data according to the sizes of facility’s animal research programs. We defined a small research program as those with less than 20 active animal research protocols, a medium research program as having 20–50 active animal research protocols, and a large research program as having more than 50 animal active research protocols.

As shown in Table 3, on the average, approximately 22 facilities each year had a small size research program (of which 16 facilities used VA IACUCs and 6 facilities used affiliated university IACUCs); 31 facilities each year had a medium size research program (of which 25 facilities used VA IACUCs and 6 facilities used affiliated university IACUCs); and 21 facilities each year had a large size research program (of which 18 facilities used VA IACUCs and 3 facilities used affiliated university IACUCs). Facilities with a small size research program had the highest lapse rate in IACUC continuing reviews, namely, 5.98%, while facilities with a large size research program had the lowest lapse rate of 3.08%. However, there were no statistically significant differences among the three groups using the Student t test with Bonferroni correction for multiple comparisons (after Bonferroni correction for multiple comparison (n = 3), in order to be considered statistical significant, *p* value needs to be < 0.017). Thus, the size of animal research programs had no correlation with the facility’s IACUC continuing review lapse rates.

**Table 3:** Lapse in institutional animal care and use committee continuing reviews according to program sizes.

Program sizes	2011	2012	2013	2014	2015	Mean (±SD)^1^
**Small (<20 protocols)**						
Number of facilities	25	22	19	22	23	21.8 (±1.6)
Lapse rates	4.8%	7.0%	13.6%	1.3%	3.2%	5.98% (±4.74%)
**Medium (20–50 protocols)**						
Number of facilities	34	33	31	28	29	31.0 (±2.5)
Lapse rates	5.7%	4.3%	1.3%	8.5%	4.7%	4.90% (±2.59%)
**Large (>50 protocols)**						
Number of facilities	15	19	24	24	23	21.0 (±3.9)
Lapse rates	5.6%	3.7%	1.3%	3.0%	1.8%	3.08% (± 1.69%)

^1^p values: 0.7543 (small vs. medium); 0.3116 (small vs. large); and 0.1611 (medium vs. large).

### Facilities with high rates of lapse in IACUC continuing reviews

Since neither the type of IACUCs used, nor the size of animal research programs had significant effects on the lapse rates of IACUC continuing review, we then focused on those facilities with a high lapse rate, namely, more than 10%. As shown in Table 4, approximately 80% of facilities each year reported no lapse in IACUC continuing reviews, approximately 6% and 14% of facilities each year reported IACUC continuing review lapse rates of >0%–10% and >10%, respectively.

**Table 4:** Number of facilities with various institutional animal care and use committee continuing review lapse rates.

Lapse rates	2011	2012	2013	2014	2015	Mean (±SD)
**0%**						
Number of facilities	59	53	63	60	61	
Percent	79.7%	71.6%	85.1%	81.1%	81.4%	79.7% (±5.0%)
**>0%-10%**						
Number of facilities	1	7	5	6	4	
Percent	1.4%	9.5%	6.6%	8.1%	5.3%	6.2% (±3.1%)
**>10%**						
Number of facilities	14	14	6	7	10	
Percent	18.9%	18.9%	8.1%	10.8%	13.3%	14.0% (±4.8%)

Analysis of facilities with a lapse rate of >10% from 2011 through 2015 revealed that 19 facilities had a lapse rate of >10% once in 5 years; 8 facilities had a lapse rate of >10% twice; 1 facilities had a lapse rate of >10% thrice; and 3 facilities had a lapse rate of >10% in 4 out of 5 years.

## Discussion

The data presented in this report demonstrate that the lapse rates in IACUC continuing reviews at VA research facilities improved from 5.6% in 2011 to 2.7% in 2015. The rate of investigators continuing research activities during the lapse also decreased from 47.2% in 2012 to 7.4% in 2015. The type of IACUCs used and the size of animal research programs appeared to have no effect in facility’s rates of lapse in IACUC continuing reviews. While approximately 80% of facilities reported no lapse in IACUC continuing reviews, approximately 14% of facilities had lapse rates of >10% each year. Some facilities appeared to be repeat offenders. For example, 4 facilities had IACUC lapse rates of >10% in at least 3 out of 5 years, suggesting a system problem in these facilities requiring remedial actions to improve their IACUC continuing reviews processes.

The major purpose of collecting performance metric data is to promote quality improvement. Each year VA research facilities were provided with their own performance metric data, including IACUC continuing review data, along with the VA national and network averages, so that each facility knows where it stands at the national and network levels (VA facilities are geographically grouped into 21 Veterans Integrated Service Networks) [11]. Thus, facilities can identify their strengths and weaknesses, and carry out quality improvement measures accordingly, as described in detail previously [11]. This might be in part responsible for the observed improvement in lapse in IACUC continuing reviews as reported here.

Performance measurement has been well recognized as an important tool for improving the quality of health care [12]. Health care providers and payers devote substantial resources to collect, analyze, and report data on providers’ performance. Our observation that lapse rates in IACUC continuing reviews improved from 5.6% in 2011 when VA started to collect IACUC performance data, to 2.7% in 2015, supports the utility of performance measurement as a potentially important tool for improving the quality of animal care and use programs. Considerable evidence suggests that postapproval monitoring can lead to improved compliance [13]. However, postapproval monitoring is a labor intensive and costly program. We believe that performance measurement of animal care and use programs can provide important information to guide facility administrators regarding where postapproval monitoring efforts should be directed.

Little is known about factors affecting the IACUC continuing review lapse rates. In the current study, we demonstrated that neither the type of IACUCs used nor the size of animal research programs had any effect on the IACUC continuing review lapse rates. On the other hand, approximately 14% of facilities each year had lapses of >10% and were largely responsible for the observed lapses in IACUC continuing reviews. In addition, some facilities were repeat offenders, suggesting that remedial actions should be directed toward these facilities. Unfortunately, our study was not designed to determine the cause(s) leading to high lapse rates, nor designed to prevent or improve lapse in IACUC continuing reviews. Therefore, we are unable to provide guidance on specific strategies to prevent or improve lapse in IACUC continuing reviews at this point. Future studies should be directed toward strategies for preventing or improving lapse in IACUC continuing reviews.

In the current study, our data collection did not make a distinction between lapse in annual continuing reviews and lapse in triennial de novo reviews. Therefore, it is not clear whether the observed lapse in IACUC continuing reviews was primarily due to annual continuing reviews or triennial de novo reviews. It is also not clear whether the observed improvement in lapse rates from 2011 through 2015 was due to improvement in annual continuing reviews or triennial de novo reviews, or both. Further studies are necessary to clarify these questions.

## Supporting Information

S1 File Lapse in IACUC continuing reviews original database. 1. Protocols lapsed in IACUC continuing reviews; and 2. Protocols audited.
(XLSX)
